# Prevalence, severity and impact of chronic pain among a representative cross‐sectional study of New Zealand high school students

**DOI:** 10.1111/jpc.16263

**Published:** 2022-11-05

**Authors:** Bridget Farrant, Simon Denny, Paul Vroegop, John Fenaughty, Terryann C Clark

**Affiliations:** ^1^ Department of Paediatrics, Faculty of Medical Health Sciences University of Auckland Auckland New Zealand; ^2^ Kidz First Centre for Youth Health Counties Manukau District Health Board Auckland New Zealand; ^3^ Mater Young Adult Health Centre Brisbane Queensland Australia; ^4^ Department of Psychological Medicine, Faculty of Medical Health Sciences University of Auckland Auckland New Zealand; ^5^ Chronic Pain Service Counties Manukau District Health Board Auckland New Zealand; ^6^ School of Counselling, Human Services and Social Work, Faculty of Education and Social Work University of Auckland Auckland New Zealand; ^7^ School of Nursing, Faculty of Medical Health Sciences University of Auckland Auckland New Zealand

**Keywords:** adolescent, chronic pain, disability, health‐care access, sexual abuse

## Abstract

**Aim:**

To report the prevalence of self‐reported chronic pain and severity among young people in New Zealand and explore the relationships between pain and mental health, substance use, socialisation and school engagement.

**Methods:**

Prevalence of self‐reported chronic pain frequency and severity are reported from an anonymous, representative cross‐sectional self‐administered health and well‐being questionnaire by students aged 12–18 years in New Zealand. Multivariable models exploring chronic pain and mental health, substance use, socialisation and school engagement are reported controlling for age, sex, ethnicity, socio‐economic status, disability and history of sexual abuse.

**Results:**

Overall, 22.8% (95% confidence interval (CI) 21.2–24.5) of young people reported chronic pain for 6 months or more, with 3.2% (95% CI 3.1–4.5) reporting severe pain weekly or more often. Females and rural adolescents were more likely to report chronic and severe pain. Asian youth reported less pain than other ethnic groups. Increased severity of pain was associated more with poorer daily functioning and socialising than with frequency of pain. Severe pain occurring weekly or more often was more common among students who had a disability (2.3% 95% CI 1.8–2.7 vs. 9.8%, 95% CI 7.2–12.5) or a history of sexual abuse (2.4% 95% 1.9–2.9 vs. 8.5%, 95% CI 6.3–10.5). Those reporting chronic pain had higher proportions of self‐reported significant depressive symptoms, lower well‐being, lower school engagement and lower access to health care, particularly for those reporting higher levels of intensity and frequency of pain.

**Conclusions:**

Chronic pain is common in adolescent populations, and has a significant association with decreased daily functioning, socialising, school engagement and mental well‐being. Adolescents with chronic pain report significant unmet health‐care needs.

## What is already known on this topic

1. Chronic pain is common in adolescents, particularly females.
2. Chronic pain can be associated with a history of childhood sexual abuse, disability, or adverse events.
3. Chronic pain in adolescence can impact on education and longer‐term vocational outcomes.

## What this paper adds

1. A high proportion of adolescents with chronic pain report that their pain is impacting on their ability to undertake everyday activities and the ability to socialise with their friends.
2. Young people with severe but less frequent pain reported more impact on their lives than young people with frequent but less severe pain.
3. Adolescents with chronic pain are more likely to report poor well‐being and being unable to access the health care they need; health‐care systems are likely underserving adolescents with chronic pain.

Chronic and recurrent pain can have a significant impact on general well‐being, school functioning,^1^ social activities and other developmentally appropriate activities.^2^, ^3^, ^4^ Persistent or chronic pain in adolescence is associated with poor quality of life and negative impacts on day‐to‐day functioning,^3^ and is a significant predictor for future persistent pain problems.^5^, ^6^, ^7^, ^8^ Chronic pain can be associated with increased rates of depression, anxiety^9^, ^10^ and substance use.^11^ The negative impact of chronic and recurrent pain in children and adolescents is multi‐factorial, bidirectional and can result in significant long‐term disability that could be mitigated with early intervention and treatment.^12^

The operational definitions of chronic or persistent pain vary; however, among adolescents, this is commonly defined as symptoms that occur at least once a week for at least 3–6 months.^13^ A landmark publication by Goodman and McGrath in 1991^14^ indicated chronic pain in children and adolescents had a point prevalence of at least 15%. In population studies across 42 countries, Gobina *et al*.^15^ found that 44% of adolescents reported any type of chronic pain (headache, backache, stomach ache and multisite pain). In New Zealand, the only population‐based data which included adolescents, found that for those aged 15–24 years, 12% of females and 8% of males reported chronic pain.^16^ More in‐depth analysis of this data source from 2006/2007 found ethnic differences with Pasifika and Asian youth half as likely to report pain compared to Māori, NZ European/Pākehā and other ethnic groups.^17^

Chronic pain has been associated with a range of adverse childhood experiences (ACEs).^9^, ^10^, ^18^, ^19^, ^20^, ^21^, ^22^ Specifically, a history of childhood sexual abuse (CSA) has been associated with increased rates of Fibromyalgia Syndrome, Irritable Bowel Syndrome^23^ and chronic pelvic pain.^24^ A recent meta‐review has reported stronger associations between CSA and self‐reported chronic pain in adults.^25^ There are limited studies exploring how CSA may be associated with chronic pain in adolescence.

This study aims to describe the epidemiology of chronic and recurrent pain among a representative community sample of adolescents in New Zealand. Specifically, we explore the associations between self‐reported frequency of chronic pain and the severity of pain with mental health and well‐being, substance use, school engagement outcomes and health‐care access. We aim to understand whether there is a dose–response relationship between the associations we examined, and whether pain intensity or pain frequency is more significant in these associations.

## Methods

We utilised the Youth19 data set, the latest wave of the Youth2000 health and well‐being surveys conducted in 2019.^26^, ^27^ Youth19 used a two‐stage cluster design to select a representative sample of secondary school students from the Northland, Auckland and Waikato regions of New Zealand. Consenting students completed the anonymous survey on handheld internet tablets in te reo Māori or English, with optional voice‐over through headphones. Ethics approval was granted by The University of Auckland Human Subjects Ethics (application #022244). In Youth19, 7721 year 9–13 students participated in the survey, and they came from 49 secondary/high schools including four kura kaupapa Māori (Māori language immersion secondary/high schools). Students ranged in ages from 12 to 19 years. Further detailed methodology can be accessed here www.youth19.ac.nz.

As part of the survey, young people were asked if they had any long‐term pain lasting 6 months or more; those that responded yes were then asked how often they got this pain (occasionally, monthly or less, once or twice a month, weekly or most days) and how severe the pain was on a scale of 1–5. The pain questions were combined in a matrix to examine the relationship between intensity of pain and frequency of pain in youth who reported chronic pain. Table 1 shows the four groupings of students experiencing chronic pain. With the addition of the group of students who do not report chronic pain, there are five groups for the analyses.

**Table 1:** Frequency and intensity of pain

	Low‐moderate pain (grade 1–3/5)	Severe pain (grade 4–5/5)
Twice monthly pain or less often	Low‐moderate/less frequent	Severe/less frequent
Weekly pain or more often	Low‐moderate/more frequent	Severe/more frequent

Three specific questions were posed for those youth who reported chronic pain; whether their pain caused difficulty with, or stopped them, doing everyday activities, communicating or socialising, or any other activity that people their age ‘can usually do’.

Outcome measures included mental health, well‐being, substance use and school engagement. See Table 2 for a full description of questions and measures.

**Table 2:** Description of variables and measures

Variable	Questions and (response options)
**Pain measures**	
Prevalence of chronic pain (6 months or more)	Do you have any long‐term pain (lasting 6 months or more), e.g., headaches, tummy pain, arms or leg pain? (yes/no)
Prevalence of frequent pain (weekly or more often)	How often do you get this pain? (Occasionally, Monthly, or less often, Once or twice a month, Weekly, Most days)
Prevalence of severe chronic pain (4 or 5 out of 5)	On a scale of 1 to 5 how bad is the pain? (1 not too bad and 5 being really bad)
Does this pain cause you difficulty with, or stop you doing …	Everyday activities that other people your age can usually do (yes/no)
	Communicating, talking, mixing with others or socialising (yes/no)
	Any other activity that people your age can usually do (yes/no)
**Emotional well‐being measures**	
High levels of depressive symptoms	Students' depressive symptoms were assessed by the Reynolds Adolescent Depression Scale‐Short Form (Mean 19.56, SE 0.07, range 10–40, Cronbach's α 0.90). This is a well‐validated 10 item instrument for measuring depression symptoms in community adolescent samples and has been demonstrated to have acceptable reliability and validity across ethnic groups within New Zealand. Young people reporting scores greater or equal to 28 were classified as experiencing high levels of depressive symptoms.^28^, ^29^
Been depressed for 2 weeks or more in the past 12 months	Assessed by a single question, ‘During the past 12 months, was there ever a time where you felt sad, blue or depressed for 2 weeks or more in a row?’ (yes/no)
Poor well‐being	The WHO‐5 is a widely used measure assessing subjective well‐being over the past 2 weeks It includes five items ‘I have felt cheerful and in good spirits’, ‘I have felt active and vigorous’, ‘I have felt calm and relaxed’ and ‘I woke up feeling fresh and rested’ and ‘My daily life has been filled with things that interest me’. Response options are a 6‐point Likert scale from ‘All of the time’ through to ‘At no time’. A score of below 13 indicates poor well‐being.^28^, ^30^
**Substance use**	
Nicotine use monthly or more often	Based on several questions assessing cigarette and e‐cigarette use and frequency. Students who indicated monthly use of either cigarettes or e‐cigarettes (that contained nicotine) were classified as monthly nicotine use.
Binge drinking (5 or more drinks within one session) Monthly or more often	Students who indicated that they drank alcohol ‘We would like to now ask some questions about alcohol. By this we mean beer, wine, spirits, pre‐mixed drinks. Have you ever drunk alcohol (not counting a few sips)?)’ and had consumed five or more alcohol drinks in one session monthly or more often were classified as binge drinking.
Marijuana use monthly or more often	Assessed by two questions: ‘Now there are some questions about marijuana. You do not have to answer if you do not want to. Remember there is no way to identify you from your answers. Have you ever used or smoked marijuana?’ (Yes/ no) and ‘In the last 4 weeks, about how often did you use marijuana?’ with response options from ‘Not at all – I do not use marijuana anymore’, ‘None in the last 4 weeks’ to ‘Several times a day’. Students who responded monthly or more often were classified as monthly marijuana use.
**School and health service engagement**	
Forgone health care	In the last 12 months, has there been any time when you wanted or needed to see a doctor or nurse (or other health‐care worker) about your health, but you were not able to? (yes/no)
Poor school achievement	How well do you do at school (how good are your school results)? (Near the top. Above middle, About the middle, Below the middle, Near the bottom). Students who reported below the middle or near the bottom were classified as poor school achievement.
Low school belonging (school engagement)	Do you feel like you are part of your school, alternative education or course? (yes/no)
Not important to go to school	How important is it to you to be at school/course every day? (Very important, Important, Not very important)
**Student demographics**	
Age, sex and ethnicity	Students self‐reported their age, sex, and ethnicity. Age was grouped into students 15 years of age and younger and students 16 years of age and older. The standard New Zealand census question was used to assess ethnicity: ‘Which ethnic group do you belong to? (you may choose as many as you need).’ 42.2% of participants identified with more than one ethnic group. To facilitate statistical analyses, discrete ethnic groups were created using the ethnicity prioritisation method, by assigning students to one ethnic group in the following order: Maori, Pacific, Asian, Other and NZ European ethnicities.
NZ Deprivation Index	Neighbourhood deprivation was measured using the New Zealand 2018 Deprivation Index. Which measures nine dimensions of deprivation including access to internet, receiving means tested benefit, household income below a threshold, unemployment rates, adults without any qualifications, home ownership, single parent families, overcrowding and damp or mouldy dwelling. This neighbourhood deprivation score was linked to student data by geocoding. During the survey, students were asked to provide their home address as to ascertain the mesh block in which they lived and residential location.
Geographical location (urban/rural)	The mesh block is a small‐area census unit of about 100 households. The categories were urban (cities, major urban areas, or large regional centres with a minimum population of 10 000 people), rural (rural and minor urbanised settlements with a population of 9999 people or fewer).
Disability	Do you have any long‐term disability (lasting 6 months or more) (e.g., sensory impaired hearing, visual impairment, in a wheelchair, learning difficulties)? (yes/no)
Sexual abuse	Have you ever been touched in a sexual way or made to do sexual things that you did not want to do? (Including sexual abuse or rape) (yes/no)

### Statistical analyses

All analyses were conducted using SAS software (SAS Institute Inc., Cary, NC) and accounted for the clustered sampling design. The prevalence estimates and their 95% confidence intervals (CIs) were derived using bivariate analyses to show simple associations between pain variables and demographic, and previously identified ‘risk’ factors. Statistically, significant differences are reported when these CIs do not overlap.

Multivariable logistic analyses show the odds ratios (ORs) between the four groups of youth reporting chronic pain, compared to those who did not report chronic pain across key health, education and well‐being outcomes. These ORs were controlled for age, gender, ethnicity, deprivation, urban/rural locality, reported sexual abuse and disability.

## Results

### Overall prevalence and demographics

As shown in Tables 3, 22.8% (95% CI 21.2–24.5) of the participants reported chronic pain for 6 months or more. For 9.9% (95% CI 8.7–11.0), this occurred weekly or more, and 6.9% (95% CI 5.9–8.0) of the sample reported severe pain (4 or 5 out 5 on a severity rating scale). Chronic pain was more common in females (26.4%, 95% CI 24.1–28.6) than males (18.4%, 95% CI 16.7–20.2) as well as more frequent and severe in females than males (see Table 3). Pain was significantly less common in Asian young people than in other ethnic groups. There was a trend for chronic pain to be more prevalent in young people living in areas of high socio‐economic deprivation, but this was not significant. Frequent pain was more common among young people in rural rather than urban settings (13.4%, 95% CI 11.1–15.8 vs. 9.7%, 95% CI 8.5–10.8).

**Table 3:** Prevalence of chronic pain by socio‐demographic variables

		Prevalence of chronic pain (6 months or more)		Prevalence of frequent pain (weekly or more often)		Prevalence of severe chronic pain (4 or 5 out of 5)	
Total		*n*	% (95% CI)	*n*	% (95% CI)	*n*	% (95% CI)
	Total	1720	22.8 (21.2–24.5)	743	9.9 (8.7–11.0)	522	6.9 (5.9–8.0)
Sex	Female	1087	26.4 (24.1–28.6)	495	12.0 (10.7–13.3)	379	9.2 (7.9–10.5)
	Male	626	18.4 (16.7–20.2)	245	7.2 (6.1–8.4)	141	4.2 (3.2–5.1)
Age	14 years and under	688	22.5 (20.8–24.3)	278	9.1 (7.9–10.3)	180	5.9 (4.8–7)
	15 years and over	1032	23.0 (21.0–25.0)	465	10.4 (9.0–11.8)	342	7.6 (6.5–8.8)
Ethnicity	Asian	266	15.2 (13.9–16.5)	92	5.3 (4.3–6.2)	73	4.2 (2.9–5.5)
	NZ European	723	23.8 (21.9–25.7)	385	12.7 (11.4–13.9)	212	7.0 (5.7–8.3)
	Maori	401	27.5 (24.7–30.2)	157	10.8 (8.3–13.3)	139	9.6 (7.8–11.3)
	Other	108	29.0 (24.7–33.3)	44	11.9 (8.3–15.5)	30	8.1 (5.6–10.5)
	Pacific	218	24.3 (20.3–28.3)	64	7.2 (5.0–9.3)	67	7.5 (5.9–9.2)
Neighbourhood deprivation	Low	465	22.2 (20.4–24.1)	226	10.8 (9.2–12.4)	133	6.4 (5–7.7)
	Med	587	21.2 (18.9–23.5)	265	9.6 (8.3–10.8)	170	6.1 (4.7–7.6)
	High	497	26.2 (23.4–29.0)	202	10.7 (8.5–12.8)	152	8.0 (6.5–9.6)
Geographical location	Urban	1257	22.2 (20.2–24.2)	546	9.7 (8.5–10.8)	364	6.4 (5.3–7.6)
	Rural	292	26.6 (23.1–30.1)	147	13.4 (11.1–15.8)	91	8.3 (6.5–10.1)
Disability	No disability	1294	20.3 (18.8–21.7)	540	8.5 (7.4–9.5)	363	5.7 (4.8–6.6)
	Disability	241	37.4 (32.6–42.2)	120	18.7 (14.7–22.6)	104	16.2 (12.5–19.9)
Sexual abuse	No sexual abuse	1119	19.2 (17.7–20.7)	469	8.1 (7.0–9.1)	308	5.3 (4.5–6.1)
	Sexual abuse	339	39.4 (35.8–43.0)	172	20.0 (17.0–23.1)	141	16.5 (13.6–19.4)

CI, confidence interval.

When the frequency and severity of pain were divided into the four groups in Table 1, the prevalence of young people reporting pain that was low to moderate in intensity occurring once or twice a month or less often was 9.0% (95% CI 8.2–9.9). For more frequent pain of low to moderate severity occurring weekly or more often, the prevalence was 6.7% (95% CI 6.0–7.4). Severe pain was less prevalent; 3.8% (95% CI 3.1–4.5) of young people reported severe pain and that occurred once or twice a month or less often, and 3.2% (95% CI 2.6–3.8) of young people reported severe pain occurring weekly or more often. Severe chronic pain irrespective of whether it was less frequent or frequent was significantly more common in females than males (see Table 4).

**Table 4:** Prevalence of frequency and severity of chronic pain by socio‐demographic variables

		None		Low to moderate pain/once or twice a month or less often		Low to moderate pain/weekly or more often		Severe pain/once or twice a month or less often		Severe pain/weekly or more often	
		*n*	% (95% CI)	*n*	% (95% CI)	*n*	% (95% CI)	*n*	% (95% CI)	*n*	% (95% CI)
	Total	5814	77.3 (75.7–79)	680	9.0 (8.2–9.9)	502	6.7 (6.0–7.4)	283	3.8 (3.1–4.5)	238	3.2 (2.6–3.8)
Sex	Female	3038	73.8 (71.6–76)	383	9.3 (7.9–10.7)	317	7.7 (6.9–8.5)	201	4.9 (4.0–5.8)	177	4.3 (3.5–5.1)
	Male	2767	81.7 (79.9–83.5)	294	8.7 (7.8–9.5)	185	5.5 (4.7–6.3)	82	2.4 (1.7–3.1)	59	1.7 (1.2–2.2)
Age	14 years and under	2366	77.7 (76–79.5)	304	10 (9–10.9)	195	6.4 (5.4–7.4)	98	3.2 (2.5–3.9)	81	2.7 (2.1–3.2)
	15 years and over	3448	77.1 (75.1–79)	376	8.4 (7.4–9.4)	307	6.9 (6.0–7.7)	185	4.1 (3.2–5.0)	157	3.5 (2.7–4.3)
Ethnicity	Asian	1486	84.8 (83.5–86.1)	129	7.4 (6.1–8.7)	64	3.7 (2.9–4.4)	45	2.6 (1.5–3.7)	28	1.6 (1–2.2)
	NZ European	2318	76.3 (74.4–78.2)	248	8.2 (7.2–9.1)	260	8.6 (7.7–9.4)	89	2.9 (2.3–3.6)	123	4.0 (3.1–5)
	Maori	1059	72.9 (70.1–75.6)	158	10.9 (9.5–12.2)	98	6.7 (5.3–8.2)	79	5.4 (4.1–6.8)	59	4.1 (2.7–5.4)
	Other	264	71.4 (67.0–75.7)	45	12.2 (9–15.3)	31	8.4 (5.8–11)	17	4.6 (2.5–6.7)	13	3.5 (1.5–5.6)
	Pacific	679	76.1 (72.3–80)	98	11.0 (8.1–13.9)	48	5.4 (3.6–7.1)	52	5.8 (4.4–7.3)	15	1.7 (1.0–2.4)
Neighbourhood deprivation	Low	1625	77.8 (76–79.6)	182	8.7 (7.8–9.6)	149	7.1 (6.1–8.2)	57	2.7 (1.8–3.6)	76	3.6 (2.8–4.5)
	Med	2184	79.0 (76.6–81.3)	229	8.3 (7.2–9.4)	184	6.7 (5.8–7.5)	89	3.2 (2.3–4.1)	80	2.9 (2.1–3.7)
	High	1400	74.1 (71.4–76.8)	202	10.7 (9.1–12.3)	136	7.2 (5.7–8.7)	86	4.6 (3.5–5.6)	66	3.5 (2.3–4.6)
Geographical location	Urban	4405	77.9 (76–79.8)	511	9.0 (8.0–10)	374	6.6 (5.8–7.4)	193	3.4 (2.6–4.2)	171	3.0 (2.4–3.6)
	Rural	805	73.7 (70.3–77.2)	102	9.3 (8.2–10.5)	95	8.7 (6.6–10.8)	39	3.6 (2.2–4.9)	51	4.7 (3.5–5.9)
Disability	No	5093	79.8 (78.4–81.2)	535	8.4 (7.5–9.2)	392	6.1 (5.4–6.9)	216	3.4 (2.7–4.0)	146	2.3 (1.8–2.7)
	Yes	404	62.9 (58.1–67.8)	78	12.1 (9.8–14.5)	56	8.7 (6.2–11.2)	41	6.4 (4–8.8)	63	9.8 (7.2–12.5)
Sexual abuse	No	4704	80.9 (79.4–82.3)	476	8.2 (7.4–9.0)	329	5.7 (4.8–6.5)	169	2.9 (2.3–3.5)	139	2.4 (1.9–2.9)
	Yes	522	61 (57.4–64.6)	95	11.1 (8.4–13.8)	98	11.4 (9.3–13.6)	69	8.1 (6.3–9.8)	72	8.4 (6.3–10.5)

CI, confidence interval.

### Disability and reported sexual abuse are associated with more frequent and severe chronic pain

About 30% (63/209) of young people with frequent and severe pain reported having a disability compared to young people with no pain where only 7.3% (404/5497) reported a disability. Young people who had a disability compared to those without, reported significantly higher chronic pain prevalence (37.4% 95% CI 32.6–42.2 vs. 20.3% 95% CI 18.8–21.7), weekly or more pain frequency (18.7% 95% CI 14.7–22.6 vs. 8.5% 95% CI 7.4–9.5) and the presence of severe chronic pain (16.2% 95% CI 12.5–19.9 vs. 5.7% 95% CI 4.8–6.6). Combining frequency and severity of reported pain; 9.8% (95% CI 7.2–12.5) of young people with disability had frequent and severe pain, compared to 2.3% (95% CI 1.8–2.7) without a reported disability (see Table 4).

About 34% (72/211) of young people with frequent and severe pain reported sexual abuse compared to young people with no pain, where only 10% (522/5226) reported sexual abuse. Those who reported sexual abuse reported significantly higher chronic pain prevalence (39.4% 95% CI 35.8–43.0 vs. 19.2% 95% CI 17.7–20.7), weekly or more pain frequency (20.0% 95% CI 17.0–23.1 vs. 8.1% 95% CI 7.0–9.1) and presence of severe chronic pain (16.5% 95% CI 13.6–19.4 vs. 5.3% 95% CI 4.5–6.1) compared to those who did not. Combining frequency and severity of reported pain; 8.4% (95% CI 6.3–10.5) of young people with a history of sexual abuse had frequent and severe pain, compared to 2.4% (95% CI 1.9–2.9) without this history (see Table 4).

### Young people's self‐reported impact on functioning

A high proportion of young people experiencing chronic pain reported a significant impact on their day‐to‐day functioning, especially when the pain was severe and frequent. About 63% (62.6%, 95% CI 56.2–69.0) of young people with frequent severe pain reported difficulty with everyday activities compared with 30.8% (95% CI 27.4–34.2) of young people with low to moderate pain occurring once or twice a month or less often. Similarly, one third (33.2%, 95% CI 26.7–39.7) of young people with frequent severe pain reported difficulty with socialising with others compared with 15% (95% CI 11.2–18.8) of young people with low to moderate pain occurring once or twice a month or less often (see Table 5).

**Table 5:** Frequency and severity of chronic pain and impact on functioning

Does pain cause you difficulty with, or stop you doing…	Low to moderate pain/once or twice a month or less often		Low to moderate pain/weekly or more often		Severe pain/once or twice a month or less often		Severe pain/weekly or more often	
	*n*	% (95% CI)	*n*	% (95% CI)	*n*	% (95% CI)	*n*	% (95% CI)
Everyday activities that other people your age can usually do	209	30.8 (27.4–34.2)	195	39.0 (33.8–44.2)	165	58.7 (53.3–64.1)	149	62.6 (56.2–69.0)
Communicating, talking, mixing with others or socialising	102	15.0 (11.2–18.8)	81	16.2 (12.8–19.6)	88	31.3 (26–36.6)	79	33.2 (26.7–39.7)
Any other activity that people your age can usually do	200	29.5 (25.6–33.3)	161	32.2 (28.3–36.1)	127	45.2 (40.3–50.1)	113	47.5 (40–54.9)

CI, confidence interval.

Overall the results suggest that severity of the chronic pain had a more significant impact on young people's lives, than the frequency (see Table 5). For both groups experiencing severe pain, approximately 60% reported difficulty with everyday activities compared with 30.8% of young people with low to moderate pain occurring monthly or less often, and 39.0% of young people with low to moderate pain occurring weekly or more often.

For mental health, reported rates of depressive symptoms and poor well‐being were greater in all groups with pain compared to young people with no chronic pain (see Table 6). For example, 51.5% of young people experiencing severe and frequent pain reported high levels of depressive symptoms compared to 20.4% of young people without chronic pain (adjusted OR (aOR) 3.1 95% CI 2.2–4.4). There was a trend for depression and poor well‐being to worsen among those reporting more severe pain followed by those with more frequent pain; however, this was not significant (see Table 6). Reported binge drinking increased with both severe and more frequent pain, but substance use increases were less consistent with nicotine and cannabis use, which showed higher use with lower levels of pain (see Table 6).

**Table 6:** Frequency and severity of chronic pain and health, education and well‐being outcomes

	None	Low to moderate pain/once or twice a month or less often		Low to moderate pain/weekly or more often		Severe pain/once or twice a month or less often		Severe pain/weekly or more often	
	%	%	aOR†	%	aOR†	%	aOR†	%	aOR†
High levels of depressive symptoms (RADS > 28)	20.4	32.8	1.7 (1.4–2.1)	39.5	2.2 (1.7–2.8)	49.6	3.0 (2.2–4.2)	51.5	3.1 (2.2–4.4)
Been depressed for 2 weeks or more in the past 12 months	33.9	48.5	1.6 (1.3–1.9)	55.6	2.0 (1.6–2.5)	64.5	2.6 (1.9–3.6)	66.7	2.6 (1.8–3.7)
Poor Well‐being (WHO5 < 13)	26.9	35.9	1.3 (1.1–1.6)	45.7	2.0 (1.6–2.6)	51.5	2.2 (1.7–2.9)	59.0	2.8 (2.1–3.7)
Nicotine use monthly or more often	8.6	10.6	1.2 (0.8–1.8)	15.1	1.6 (1.2–2.2)	17.1	1.7 (1.1–2.5)	14.5	1.3 (0.9–2.0)
Binge drinking (5 or more drinks within one session) Monthly or more often	18.6	20.4	1.0 (0.7–1.4)	27.3	1.3 (1.0–1.8)	31.2	1.7 (1.1–2.7)	35.4	1.7 (1.2–2.3)
Marijuana use monthly or more often	7.9	8.9	0.9 (0.6–1.2)	15.1	1.9 (1.3–2.7)	17.7	2.0 (1.2–3.1)	12.8	1.0 (0.6–1.6)
Forgone health care	17.0	26.8	1.6 (1.2–2.1)	35.1	2.5 (2.0–3.2)	43.1	2.8 (2–3.9)	47.8	3.4 (2.4–4.9)
Poor school achievement (below middle)	7.6	12.3	1.4 (1.0–2.1)	10.6	1.3 (0.9–1.8)	12.9	1.4 (0.8–2.5)	18.3	2.5 (1.6–4)
Poor school engagement	12.6	16.4	1.4 (1.0–1.8)	17.3	1.4 (1.0–1.9)	20.4	1.8 (1.2–2.7)	19.6	1.5 (1–2.4)
Not important to go to school	3.9	4.7	1.1 (0.7–1.7)	7.2	2.3 (1.3–4.0)	8.6	1.7 (0.9–3.0)	10.6	1.4 (0.8–2.7)

† Controlling for age, gender, ethnicity, NZDep, Urban/rural, sexual abuse and disability.

aOR, adjusted odds ratio; RADS, reynolds adolescent depression scale; WHO5, world health organisation ‐ wellbeing index 5.

### School engagement and achievement

Students with chronic pain were more likely to report poor school engagement than those without chronic pain (see Table 6). The association between chronic pain and the student‐reported importance of going to school was inconsistent. Self‐rated poor school achievement (below middle) was significantly more likely in those with severe and chronic pain compared to no pain (aOR 2.5 95% CI 1.6–4).

### Forgone health care

Young people with chronic pain reported significantly greater rates of forgone health care (not being able to see health‐care workers when needed for their health in the last 12 months) than those without chronic pain (Table 6). Almost 50% of young people with frequent and severe pain reported forgone care versus 27% among those with no pain (aOR 3.4 95% CI 2.4–4.9).

## Discussion

Chronic pain among adolescents in New Zealand is common, with one in five (23%) reporting chronic pain, and a third of this group reporting severe pain. Consistent with previous research,^15^ chronic pain is more common among females and those living in rural settings, and less common among Asian adolescents. Young people with severe but less frequent pain reported more impact on their lives than young people with frequent but less severe pain, which confirms some previous findings.^31^ Similar to previous studies,^32^ our findings show a strong association between frequent and severe pain and self‐reported disability.

Our findings show that there are strong associations between sexual abuse and chronic pain in adolescence. It is not clear whether this association is mediated by previously identified associations such as depression, PTSD, poor physical health and substance abuse, although it has been suggested that childhood abuse and depression independently contributed to pain reporting in adults.^33^

The findings of poor mental health associated with more frequent and serve pain are consistent with other studies^9^, ^10^, ^31^ highlighting the importance of assessing the mental health of young people with chronic pain. While our findings found a relationship between increasing severity and frequency of chronic pain and alcohol use, the relationship between increasing severity and frequency of chronic pain and marijuana use and nicotine use was less clear. This is an area for future research given the public discussion about use of marijuana for pain management.

Research indicates that the negative association of pain with school performance goes beyond the issues associated with school absenteeism and achievement, to include the disruptions produced by cognitive challenges. As Gorodzinsky *et al*.^1^ argue, pain not only affects academic achievement, but also social development like participating in sports and other extracurricular activities. Our results showing that students with pain, especially those who reported more frequent or severe pain, were more likely to report an impact on everyday functioning and less likely to say that they felt like a part of their school or education institution, emphasises the importance of considering the implications of pain beyond academic achievement.

Young people with chronic pain report high rates of inability to access health care when needed. Almost 50% of young people with severe and frequent pain report that they have been unable to access health care when required during the previous 12 months. These findings likely reflect that health services are underserving the needs of adolescents with chronic and recurrent pain. This may also signal a lack of available local expertise and services in primary and secondary care to address pain from an adolescent developmental approach. There may also be a mismatch between what adolescents and their families understand the treatment options are for chronic pain; a widely held mechanistic and biomedical understanding of persistent pain being caused by body damage that will be ‘cured’ with a pharmacological or surgical approaches, versus an evidence‐based biopsychosocial‐cultural‐spiritual framework focused on improving function and pain management, supported by psychological approaches. This may lead to disappointment when young people's expectations for health care (such as surgery or medication, which may have limited efficacy in chronic pain) are not met, or when care offered (such as physical therapy or psychological approaches) are not seen as relevant or taking their pain seriously. Services that are responsive, engaging and work towards solutions that reduce the physical and psychosocial impacts of pain on young people are required.

There are several major limitations to this study, determining causation of the reported associations cannot be made. A sampling limitation is that adolescents with chronic pain tend to have significantly poorer school attendance and may therefore our results may underestimate the true population prevalence and severity of pain. A further limitation is that this was a self‐report survey, and we are unable to validate some aspects such as school achievement or what students understood by ‘long‐term disability’. Finally, the survey did not specifically ask questions about the sites of pain; however, chronic pain in different bodily regions frequently co‐occurs.^15^

## Conclusions

Chronic and severe pain is common among this representative sample of New Zealand adolescents with significant negative associations with mental health, education and social functioning. Yet it is likely poorly assessed, under‐treated with sub‐optimal management. Ultimately, these findings suggest the importance of improving access to health care and exploring evidence‐based models of care for adolescents with chronic and severe pain to mitigate poor health and social outcomes.
